# Design, Synthesis, Theoretical Study, and Antioxidant Activity of Aromaticity-Extended Resveratrol Derivatives Incorporating Chalcogen

**DOI:** 10.3390/ijms26125872

**Published:** 2025-06-19

**Authors:** Sangwon Ko, Hyun Min Lim, Yeonho Song, Hyonseok Hwang, Jeong Tae Lee

**Affiliations:** 1 Transportation Environmental Research Department, Korea Railroad Research Institute, Uiwang 16105, Republic of Korea; sko@krri.re.kr; 2 Department of Chemistry and Institute of Applied Chemistry, Hallym University, Chuncheon 24252, Republic of Korea; tnghals@naver.com; 3 Department of Chemistry and Institute for Molecular Science and Fusion Technology, Kangwon National University, Chuncheon 24341, Republic of Korea; syh6076@naver.com

**Keywords:** antioxidant activity, scavenging assay, resveratrol derivatives, benzofuran, benzothiophene, benzoselenophene

## Abstract

Naturally occurring antioxidants have attracted significant research interest, owing to their radical scavenging ability that can be improved via structural modifications. In this study, aromaticity-extended resveratrol analogues (**3**–**5**) containing chalcogens were designed and synthesized using ring closure and Horner–Wadsworth–Emmons reactions. The antioxidant activities of the derivatives were evaluated using 2,2′-azino-bis(3-ethylbenzothiazoline-6-sulfonic acid) (ABST) assay. All resveratrol derivatives (**3**–**5**) exhibited higher radical scavenging activities than resveratrol **1** and analogue **2**, with benzoselenophene-conjugated derivative **5** demonstrating the highest activity. The improved antioxidant performance of the resveratrol derivatives was attributed to the extended π conjugation resulting from the incorporation of fused rings, benzoheteroles. Additionally, the integration of benzoheteroles into resveratrol contributed to an efficient reduction in HOMO-LUMO gaps. This study demonstrates that aromaticity extension by introducing benzofuran, benzothiophene, and benzoselenophene is a feasible strategy for improving the antioxidant activity of naturally occurring oxidants.

## 1. Introduction

**Figure 1:**
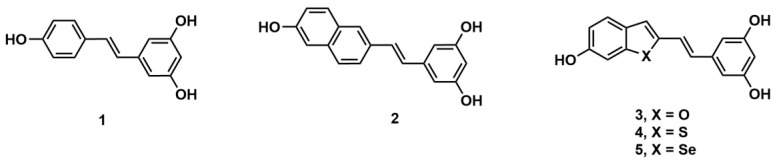
Structure of resveratrol **1** and derivatives **2**–**5**.

Reactive oxygen species (ROS) induce oxidative damage to cells, particularly targeting DNA, proteins, and lipids. This oxidative stress is a key factor in cancer development, as DNA damage can result in mutations, abnormal cell growth, and disrupted differentiation [1]. Antioxidant components in enzymes and food scavenge ROS to hinder radical chain reactions, thereby reducing lipid peroxidation and cellular damage. To prevent cellular damage, the body has a complex network of antioxidant defenses that includes enzymatic and non-enzymatic antioxidants; however, an imbalance of antioxidants and ROS in the body leads to oxidative stress, which can cause cellular damage and a number of diseases [2,3]. Therefore, adequate supplementation with exogenous antioxidants is essential to protect the body from diseases. Exogenous antioxidants are classified as either synthetic or natural. Synthetic antioxidants, such as butylated hydroxyanisole (BHA) and butylated hydroxytoluene (BHT), are widely used because of their low costs and high antioxidant activities [4]. However, excessive use and improper application of synthetic antioxidants have been associated with side effects such as cancer [5]. Consequently, research has focused on deriving antioxidants from natural sources with relatively few side effects [5,6]. Plant-derived natural antioxidants, such as phenolic compounds, are widely used as additives in food, medicine, and cosmetics because of their ability to inhibit oxidative stress caused by lipid oxidation and ROS. Resveratrol (3,5,4′-trihydroxy-*trans*-stilbene) (**1**) (Figure 1) is a naturally occurring phenolic stilbenoid found mainly in grapes, mulberries, and peanuts. Previous studies have shown that resveratrol **1** exhibits antioxidant, anticancer, anti-inflammatory, antidiabetic, and cardioprotective properties [7]. The antioxidant activity of resveratrol can be attributed to several chemical and biological mechanisms, including the stabilization of free radicals, inhibition of lipid peroxidation, and upregulation of endogenous antioxidant enzymes [7,8]. Notably, compared to resorcinol, the hydroxyl group of the phenol moiety in resveratrol has been reported to exhibit superior hydrogen transfer and peroxyl radical scavenging activities [9]. To improve biological activity of phenoxy radicals, structural modification via elongating conjugation and encapsulation with a bovine serum albumin (BSA)–caffeic acid (CA) conjugate has been suggested [10,11,12,13]. The extended π conjugation of phenoxy radicals has been proposed as a strategy for stabilizing their resonance and enhancing their scavenging properties. In particular, aromaticity extension can contribute to the elongation of π-conjugated systems, maximizing delocalization and lowering the dissociation energy of phenolic O–H bonds [10]. Fused ring systems facilitate charge delocalization in cyclic conjugation, making them promising approaches to achieve the aromaticity extension strategy [14]. A previous study on π-extended resveratrol derivatives, including compound **2** with fused benzene rings, revealed enhanced ABTS radical scavenging activities compared to resveratrol [8].

In this study, we designed resveratrol derivatives **3**–**5** with extended aromaticity by introducing fused rings containing chalcogens such as benzofuran, benzothiophene, and benzoselenophene (Figure 1). In each designed compound, the chalcogen atoms contribute two π electrons, resulting in a total of ten π electrons. This satisfies Hückel’s rule for aromaticity, with the π-electrons delocalized throughout the fused ring systems. The synthetic routes to compounds **3**–**5** are discussed, and their antioxidant activities are compared in relation to the extended aromaticity, including a bicyclic π system found in resveratrol derivative **2** [8]. Although benzofuran and benzothiophene do not inherently possess significant antioxidant activity**,** their derivatives with phenolic hydroxyl groups, capable of scavenging free radicals or donating hydrogen atoms, can exhibit antioxidant properties. Thus, benzofuran and benzothiophene have been used as building blocks for the synthesis of heterocyclic compounds that show biological activities [15,16]. Selenium is an essential trace element that plays many biological roles in the body, including antioxidation, antitumor, and anti-inflammatory activities. In particular, selenophene derivatives have shown antioxidant activities, such as the neutralization and scavenging of free radicals and other ROS [9,16,17]. Therefore, this study aimed to propose synthetic routes and compare the radical scavenging activities of new resveratrol derivatives with fused heterocyclic rings.

## 2. Results and Discussion

### 2.1. Preparation of the Resveratrol Derivatives

**Scheme 1:**
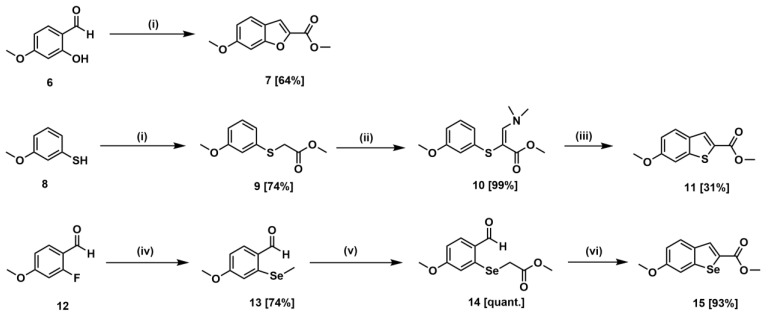
Synthesis of benzofuran, benzothiophene, and benzoselenophene derivatives **7**, **11**, and **15**. Reagents and conditions: (**i**) methyl bromoacetate, K_2_CO_3_, reflux; (**ii**) *N*,*N*-dimethylformamide dimethyl acetal, 80 °C; (**iii**) iodine, CHCl_3_, reflux; (**iv**) dimethyl diselenide, dithiothreitol, DMF, DBU, 80 °C; (**v**) methyl bromoacetate, reflux; (**vi**) K_2_CO_3_, CH_3_CN, reflux.

The synthesis of resveratrol derivatives **3**, **4**, and **5** commenced with the formation of benzofuran, benzothiophene, and benzoselenophene derivatives **7**, **11**, and **15**, respectively (Scheme 1). Derivative **2** was synthesized according to the previously reported method [8]. Treatment of 2-hydroxy-4-methoxybenzaldehye **6** with methyl bromoacetate in the presence of K_2_CO_3_ afforded methyl 6-methoxybenzofuran-2-carboxylate **7** in 64% yield. In a Rap–Stoermer reaction to synthesize benzofuran derivatives, the use of CH_3_CN as the solvent resulted in a higher yield of compound **7** (64%) compared to dimethylformamide (DMF), which provided a yield of 27%. For the synthesis of 6-methoxybenzo[*b*]thiophene-2-carboxylate **11**, 3-methoxybenzenethiol **8** was used as the starting material. The thiol moiety of 3-methoxybenzenethiol **8** was transformed into thioacetate **9** in 74% yield using methyl bromoacetate and K_2_CO_3_. To generate an enamine fragment at the *α* carbon of the carbonyl group, compound **9** underwent a condensation reaction with DMF-dimethylformamide dimethyl acetal (DMA), yielding dimethylaminomethylene derivative **10**. A ring closure reaction of **10**, performed using iodine in chloroform, afforded 6-methoxybenzo[*b*]thiophene-2-carboxylate **11** in 31% yield. To synthesize benzoselenophene derivative **15**, 2-fluoro-4-methoxybenzaldehyde **12** was treated with a mixture of dimethyl diselenide and dithiothreitol in DMF. In an addition–elimination reaction, methyl selenoate served as a more effective nucleophile than ethyl selenoate because of its lower steric hindrance, resulting in a 74% yield of 4-methoxy-2-(methylselanyl)benzaldehyde **13**. Under reflux conditions, with excess methyl bromoacetate, 4-methoxy-2-(methylselanyl)benzaldehyde quantitatively transformed into methyl 2-((2-formyl-5-methoxyphenyl)selanyl)acetate **14**. The formation of α carbon in selanyl acetate **14** facilitated cyclization and dehydration under K_2_CO_3_, affording 6-methoxybenzo[*b*]selenophene-2-carboxylate **15** in 93% yield.

**Scheme 2:**
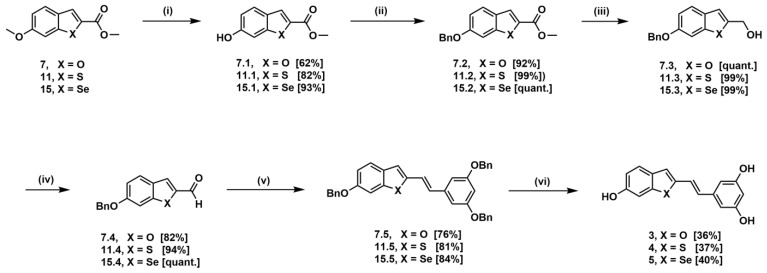
Synthesis of aromaticity extended resveratrol derivatives **3**, **4**, and **5**. Reagents and conditions: (**i**) BBr_3_, DCM, room temp.; (**ii**) benzyl bromide, K_2_CO_3_, acetone, room temp.; (**iii**) LiAlH_4_, THF, room temp.; (**iv**) 2-iodoxybenzoic acid, DMSO, 90 °C; (**v**) NaH, diethyl 3,5-bis(benzyloxy)benzylphosphonate, DMF, room temp.; (**vi**) *N*,*N*-dimethylaniline, DCM, AlCl_3_, room temp.

Compound **7**, **11**, and **15**, obtained through cyclization, underwent the same synthetic route to afford π-extended target molecules **3**, **4**, and **5** as shown in Scheme 2. The methoxy substituents of compounds **7**, **11**, and **15** were replaced with benzyl-protecting groups, allowing for simultaneous deprotection with other benzyl groups in the final step. BBr_3_ was used for the demethylation of compounds **7**, **11**, and **15** to produce **7.1**, **11.1**, and **15.1** with yields ranging from 62% to 93%. Subsequent protection with benzyl bromide afforded carboxylates **7.2**, **11.2**, and **15.2** in yields ranging from 92% to quantitative. To introduce conjugated vinyl benzene, carboxylates **7.2**, **11.2**, and **15.2** were transformed to carbaldehydes **7.4**, **11.4**, and **15.4**, respectively, through quantitative lithium aluminum hydride (LAH) reduction followed by 2-iodoxybenzoic acid (IBX) oxidation, achieving yields ranging from 82% to quantitative. During the oxidation of (6-(benzyloxy)benzofuran-2-yl)methanol **7.3**, IBX afforded carboxaldehyde **7.4** in 82% yield, whereas MnO_2_ showed no reactivity and pyridinium dichromate (PDC) resulted in a lower yield of 40%.

To conjugate *E*-alkene with carbaldehydes **7.4**, **11.4**, and **15.4**, a Horner–Wadsworth– Emmons reaction was performed with diethyl 3,5-bis(benzyloxy)benzylphosphonate and NaH [8]. Using DMF as the solvent, the olefination of carbaldehyde **7.4** formed (*E*)-6-(benzyloxy)-2-(3,5-bis(benzyloxy)styryl)-benzofuran **7.5** in 76% yield, which is significantly higher than the 20% yield obtained using THF as the solvent. Under identical conditions, the Horner–Wadsworth–Emmons reaction produced (*E*)-6-(benzyloxy)-2-(3,5-bis(benzyloxy)styryl)benzo[*b*]thiophene **11.5** and (*E*)-6-(benzyloxy)-2-(3,5-bis(benzyloxy)styryl)benzo[*b*]selenophene **15.5** in 81% and 84% yields, respectively. Finally, deprotection of benzyl groups using AlCl_3_ afforded π-extended resveratrol derivatives **3**, **4**, and **5** in 36%, 37%, and 40% yields, respectively, whereas BBr_3_ did not work for deprotection.

### 2.2. Antioxidant Activity

**Figure 2:**
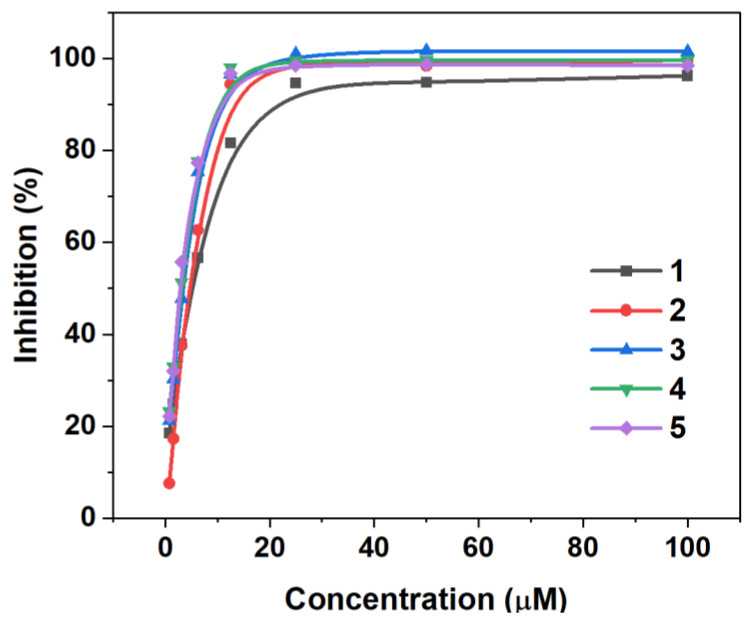
Radical scavenging activities of resveratrol **1** and aromaticity-extended resveratrol derivatives (**2**–**5**) in ABTS assay.

To evaluate the antioxidant activities of the designed compounds, an ABTS scavenging assay was used [18]. An ABTS assay is efficient for estimating antioxidant capacity and involves electron transfer reduction by antioxidants. The ABTS assay measures the reduction of a blue–green ABTS radical cation, which shows a decrease in absorbance at 734 nm upon interaction with antioxidants. Free radical scavenging activities of compounds **1**–**5**, determined using the ABTS assay, are presented in Figure 2. Radical inhibition activities of derivatives **2**–**5** increased sharply as the concentration increased, exceeding that of resveratrol **1**, although compound **2** was the least effective at low concentrations. Half-maximal inhibitory concentration (IC_50_) values indicate the concentration of the sample required to reduce the initial free radical concentration by 50%. As listed in Table 1, derivatives **2**, **3**, **4**, and **5** exhibited IC_50_ values of 4.16 ± 0.12, 3.39 ± 0.59, 3.02 ± 0.30, and 2.75 ± 0.30 μM, respectively. These results indicate that compounds **2**–**5** possess superior scavenging activities relative to resveratrol **1**, which has an IC_50_ of 5.15 ± 0.35 μM. The enhanced antioxidant activities of resveratrol derivatives **2**–**5** as compared to those of resveratrol **1** can be attributed to the improved stabilization of radical species such as phenoxy radicals via delocalization of the π electrons by extended and planar π conjugation. In comparison to resveratrol analogue **2** fused with the benzene ring, the derivatives **3**, **4**, and **5** fused with heterocyclic compounds exhibited enhanced ABTS radical scavenging activity. Notably, the IC_50_ values against ABTS radicals improved with the atomic number of the chalcogen in the benzoheteroles **3**, **4**, and **5**. While the high electronegativity of oxygen in the benzofuran moiety can reduce π delocalization, the larger and more polarizable selenium enhances π delocalization. In addition to ABTS, DPPH scavenging assays were also used for compounds **1**, **3**, **4**, and **5** [19]. According to Figure S1 and Table S1, resveratrol **1** displayed negligible antioxidant activity in the DPPH assay, exhibiting an IC_50_ value exceeding 100 μM. In contrast, derivatives **3**, **4**, and **5** showed significant antioxidant properties, exhibiting IC_50_ values of 40.20 ± 0.89, 46.52 ± 3.42, and 36.71 ± 1.41 μM, respectively. While the derivative **3,** containing benzofuran, showed a better IC_50_ value than that of derivative **4** with benzothiophene in the DPPH assay, derivative **5** demonstrated the highest antioxidant activity in both the ABTS and DPPH assays.

Extended conjugation in resveratrol derivatives **2**–**5** was further explored by performing calculations using B3LYP/6-31G+(d,p) in the gas phase and MeOH, the results of which are presented in Table 2. HOMO-LUMO gaps of derivatives **2**–**5** in both the gas phase and MeOH were smaller than that of resveratrol **1**, with similar energy gaps observed among derivatives **3**–**5**. Compared to naphthalene analogue **2**, derivatives **3**–**5** with benzoheteroles showed smaller energy gaps. These theoretical observations align with the improved ABTS radical scavenging activity of compounds **3**–**5**. Benzofuran, benzothiophene, and benzoselenophene are known to have planar structures that contribute to the extension of π conjugation [14,20,21]. In addition, the excitation in the UV spectral energies were observed to shift toward lower energies as the size of the chalcogens of the benzoheteroles increases, resulting in a systematic decrease in the HOMO–LUMO gap [14]. Therefore, the findings from the aforementioned literature also provide a rationale for the superior antioxidant activities of derivatives **3**–**5** compared to the parent, resveratrol **1**. Especially, benzoselenophene-conjugated derivative **5**, exhibiting the highest antioxidant activity in the ABTS assay, was identified as the most efficient antioxidant among the studied derivatives.

## 3. Materials and Methods

### 3.1. Materials

All chemicals were obtained from Aldrich (St. Louis, MO, USA), TCI (Tokyo, Japan), Alfa Aesar (Heysham, UK), Junsei (Tokyo, Japan), Samchun (Seoul, Republic of Korea), and Burdick & Jackson (Muskegon, MI, USA) and were used without further purification. All solvents used for reactions were freshly distilled using appropriate dehydrating agents under argon or purchased as anhydrous solvents. All solvents used for chromatography were purchased and used without further purification. ^1^H-NMR spectra were recorded on a Varian Mercury (Varian, CA, USA) 300 MHz FT-NMR and 75 MHz for ^13^C, with the chemical shift (δ) reported in parts per million (ppm) downfield relative to TMS and the coupling constants (*J*) quoted in Hz. Peak splitting patterns were abbreviated as s (singlet), d (doublet), t (triplet), q (quartet), dd (doublet of doublet), and m (multiplet). CDCl_3_, CD_3_OD, (CD_3_)_2_CO, or DMSO-*d*_6_ was used as a solvent, and TMS (tetramethylsilane) was used an internal standard in NMR measurement. Mass spectra were recorded using JEOL’s JMS-700 (JEOL, Tokyo, Japan)/Agilent-5977E/Agilent-7802A (Agilent, Santa Clara, CA, USA) spectrometer. High-resolution mass spectra were recorded on a JMS-700 (JEOL, Tokyo, Japan) spectrometer. Reactions were monitored by thin-layer chromatography (TLC) on silica gel 60 F_254_ (Merck, Darmstadt, Germany) plates and visualized by UV light (254 nm) or stained with *p*-anisaldehyde and phosphomolybdic acid. Chromatographic purification was carried out using Silica gel 60 (230-400 mesh, Merck, Darmstadt, Germany).

### 3.2. Syntheses

#### 3.2.1. Methyl 6-Methoxybenzofuran-2-carboxylate (**7**)

K_2_CO_3_ (2.73 g, 19.72 mmol) and methyl bromoacetate (0.75 mL, 7.89 mmol) were added to a stirred solution of compound **6** (1.0 g, 6.57 mmol) in CH_3_CN (65 mL). The reaction mixture was refluxed for 4 h under argon. After the reaction was completed, the reaction mixture was brought to room temperature, filtered through a Celite^®^ pad, and washed with CH_3_CN (15 mL). Then, the filtrate was concentrated under reduced pressure. The residue was purified by column chromatography (EtOAc/hexane = 1/8) to yield compound **7** (0.88 g, 4.27 mmol, 64.7%) as a white solid. ^1^H NMR (300 MHz, CDCl_3_) δ: 3.84 (s, 3H), 3.93 (s, 3H), 6.91 (dd, *J* = 8.7, 2.1 Hz, 1H), 7.02 (d, *J* = 2.1 Hz, 1H), 7.43 (s, 1H), 7.50 (d, *J* = 8.7 Hz, 1H); ^13^C NMR (75 MHz, CDCl_3_) δ: 52.2, 55.5, 95.4, 113.8, 114.1, 119.9, 122.9, 144.3, 156.8, 159.7, 160.3; GC-MS *m*/*z* 206 (M^+^, base).

#### 3.2.2. Methyl 6-Hydroxybenzofuran-2-carboxylate (**7.1**)

A stirred solution of compound **7** (0.4 g, 1.94 mmol) in anhydrous CH_2_Cl_2_ (12 mL) was cooled to −20 °C under an argon atmosphere. BBr_3_ (1.0 M in CH_2_Cl_2,_ 0.4 mL, 4.27 mmol) was then added dropwise. After 30 min of stirring at −20 °C, the reaction mixture was brought to room temperature and stirred for an additional 3 h. Then, the reaction mixture was cooled again to 0 °C, and MeOH (2 mL) was slowly added to quench the excess BBr_3_, followed by stirring for 10–15 min. The reaction mixture was extracted with CH_2_Cl_2_ (3 × 30 mL), and the combined organic layers were washed with distilled H_2_O (2 × 25 mL) and brine (25 mL), dried over anhydrous Na_2_SO_4_, and concentrated in vacuo. The residue was purified by column chromatography (EtOAc/hexane = 1/2) to afford compound **7.1** (0.23 g, 62.2%) as a white solid. ^1^H NMR (300 MHz, CD_3_OD) δ: 3.98 (s, 3H), 6.82 (dd, *J* = 8.7, 2.1 Hz, 1H), 6.90 (d, *J* = 2.1 Hz, 1H), 7.47 (s, 1H), 7.49 (d, *J* = 8.7 Hz, 1H); ^13^C NMR (75 MHz, CD_3_OD) δ: 52.7, 98.6, 115.3, 115.7, 120.7, 124. 5, 145.3, 158.8, 160.0, 161.6; GC-MS *m*/*z* 192 (M^+^, base).

#### 3.2.3. Methyl 6-(Benzyloxy)benzofuran-2-carboxylate (**7.2**)

K_2_CO_3_ (0.80 g, 5.78 mmol) and benzyl bromide (0.55 mL, 4.62 mmol) were added to a solution of compound **7.1** (0.74 g, 3.85 mmol) in acetone (10 mL) under an argon atmosphere at room temperature. The solution was refluxed for 4 h with stirring, and the reaction mixture was brought to room temperature, filtered through a Celite^®^ pad, and washed with acetone (15 mL). The filtrate was concentrated in vacuo. The residue was dissolved in EtOAc (50 mL) and washed with distilled H_2_O (2 × 15 mL) and brine (15 mL), dried over anhydrous Na_2_SO_4_, and concentrated in vacuo. The residue was purified by column chromatography (EtOAc/hexane = 1/4) to afford compound **7.2** (1.00 g, 3.54 mmol, 92.6%) as a white solid. ^1^H NMR (300 MHz, CDCl_3_) δ: 3.94 (s, 3H), 5.10 (s, 2H), 7.00 (dd, *J* = 8.7, 2.1 Hz, 1H), 7.10 (d, *J* = 2.1 Hz, 1H), 7.31–7.44 (m, 6H), 7.52 (d, *J* = 8.7 Hz, 1H); ^13^C NMR (75 MHz, CDCl_3_) δ: 52.5, 71.0, 97.5, 114.2, 114.8, 120.7, 123.2, 127.6, 128.2, 128.8, 136.7, 145.1, 157.2, 159.8, 159.9; GC-MS *m*/*z* 282 (M^+^, base).

#### 3.2.4. (6-(Benzyloxy)benzofuran-2-yl)methanol (**7.3**)

LiAlH_4_ (2.0 M in THF, 2.71 mL, 5.31 mmol, 1.5 eq.) was added dropwise to a stirred solution of compound **7.2** (1.0 g, 3.54 mmol) in THF (5 mL) at 0 °C under an argon atmosphere. After completion of addition, the reaction mixture was brought to room temperature and stirred for an additional 90 min. After the reaction was completed, the reaction mixture was cooled to 0 °C, and the excess LiAlH_4_ was quenched by aq. sat. K_2_CO_3_ solution (1 mL). Then, the resulting mixture was filtered through a Celite^®^ pad and washed with EtOAc (20 mL). The filtrate was dried over Na_2_SO_4_ and concentrated in vacuo to afford compound **7.3** (0.90 g, quantitative) as a white solid. ^1^H NMR (300 MHz, CDCl_3_) δ: 1.65 (br s, 1H), 4.70 (s, 2H), 5.08 (s, 2H), 6.56 (s, 1H), 6.92 (dd, *J* = 8.1, 2.1 Hz, 1H), 7.04 (d, *J* = 2.1 Hz, 1H), 7.30–7.44 (m, 6H); ^13^C NMR (75 MHz, CDCl_3_) δ: 52.3, 71.0, 97.5, 114.2, 114.8, 120.7, 123.2, 127.6, 128.2, 128.8, 136.7, 145.1, 157.2, 159.8; GC-MS *m*/*z* 254 (M^+^, base).

#### 3.2.5. 6-(Benzyloxy)benzofuran-2-carbaldehyde (**7.4**)

A mixture of compound **7.3** (0.29 g, 1.14 mmol) and IBX (2-iodoxybenzoic acid, 0.48 g, 1.71 mmol) in DMSO (5 mL) was stirred for 2 h at 90 °C under an argon atmosphere. After the addition of EtOAc (40 mL), the organic layer was washed with aq. sat. NaHCO_3_ solution (15 mL) and distilled H_2_O (3 × 15 mL) and brine (2 × 15 mL) and dried over anhydrous Na_2_SO_4_. Solvent evaporation under reduced pressure yielded compound **7.4** (0.24 g, 82.7%) as a yellow solid. ^1^H NMR (300 MHz, CDCl_3_) δ: 5.13 (s, 2H), 7.07 (dd, *J* = 8.7, 2.1 Hz, 1H), 7.09 (d, *J* = 2.1 Hz, 1H), 7.03–7.47 (m, 6H), 7.59 (d, *J* = 8.7 Hz, 1H), 9.72 (s, 1H); ^13^C NMR (75 MHz, CDCl_3_) δ: 70.9, 97.3, 115.4, 117.7, 120.4, 123.9, 127.4, 128.2, 128.6, 136.3, 152.8, 157.9, 160.8, 178.4; GC-MS *m*/*z* 252 (M^+^, base).

#### 3.2.6. (*E*)-6-(Benzyloxy)-2-(3,5-bis(benzyloxy)styryl)benzofuran (**7.5**)

Diethyl 3,5-bis(benzyloxy)benzylphosphonate (0.80 g, 1.82 mmol) in DMF (4 mL) was added dropwise to a suspension of NaH (60% in mineral oil, 0.13 g, 5.47 mmol) in DMF (3 mL) at 0 °C and stirred for 20~30 min. Compound **7.4** (0.46 g, 1.82 mmol) in DMF (5 mL) was transferred dropwise to the above mixture under an argon atmosphere. The resulting mixture was brought to room temperature and stirred for 3 h. After the reaction was completed, distilled H_2_O (5 mL) was added to the reaction mixture slowly to quench excess NaH. The resulting mixture was extracted with EtOAc (3 × 30 mL). The combined organic layer was washed with distilled H_2_O (2 × 15 mL) and brine (15 mL), dried over anhydrous Na_2_SO_4_, and concentrated in vacuo. The residue was purified by column chromatography (EtOAc/hexane = 1/8) to afford compound **7.5** (0.75g, 76.5%) as a yellow solid. ^1^H NMR (300 MHz, CDCl_3_) δ: 5.04 (s, 4H), 5.09 (s, 2H), 6.54 (t, *J* = 2.2 Hz, 1H), 6.57 (s, 1H) 6.74 (d, *J* = 2.2 Hz, 2H) 6.88 (d, *J* = 15.9 Hz, 1H), 6.90 (dd, *J* = 8.4, 2.2 Hz, 1H), 7.05 (d, *J* = 2.2 Hz, 1H), 7.11 (d, *J* = 15.9 Hz, 1H), 7.35–7.45 (m, 16H); ^13^C NMR (75 MHz, CDCl3) δ: 70.2, 70.6, 96.9, 101.9, 105.4, 105.8, 112.5, 117.0, 120.9, 122.6, 127.3, 127.4, 127.9, 128.5, 128.7, 136.7, 136,8, 138.7, 154.1, 155.8, 157.4, 160.0; MS (EI^+^) *m*/*z* 538 (M^+^, base), 447, 356, 265.

#### 3.2.7. (*E*)-5-(2-(6-Hydroxybenzofuran-2-yl)vinyl)benzene-1,3-diol (**3**)

AlCl_3_ (4.34 g, 32.53 mmol) was added to a mixture of compound **7.5** (0.73 g, 1.36 mmol) and *N*,*N*-dimethylaniline (2.96 mL, 24.40 mmol) in DCM (10 mL) under an argon atmosphere and stirred for 3 h at room temperature. After the reaction was completed, 1M HCl (10 mL) was added to the mixture dropwise at 0 °C and stirred for 10 min. The reaction mixture was extracted with EtOAc (3 × 30 mL). The combined organic layer was washed with aq. sat. NaHCO_3_ solution (15 mL) and distilled H_2_O (2 × 15 mL) and brine (15 mL) dried over anhydrous Na_2_SO_4_, and concentrated in vacuo. The residue was purified by column chromatography (CH_2_Cl_2_/MeOH = 97/3), yielding **3** (0.13 g, 36.1%) as a yellow solid. ^1^H NMR (300 MHz, (CD_3_)_2_CO) δ 8.58 (br s, 1H), 8.33 (br s, 2H), 7.35 (dd, *J* = 8.4, 0.8 Hz, 1H), 7.03 (d, *J* = 16.0 Hz, 1H), 6.98 (d, *J* = 16.0 Hz, 1H), 6.94 (d, *J* = 2.0 Hz, 1H) 6.77 (dd, *J* = 8.4, 2.0 Hz, 1H), 6.71 (d, *J* = 0.8 Hz, 1H), 6.57 (d, *J* = 2.0 Hz, 1H), 6.31 (t, *J* = 2.0 Hz, 1H); ^13^C NMR (75 MHz, (CD_3_)_2_CO) δ 98.5, 103.7, 106.2, 113.2, 117.5, 121.9, 122.7, 129.6, 139.8, 154.9, 157.0, 157.1, 159.6; MS (EI^+^) *m*/*z* 268 (M^+^, base); HRMS *m*/*z* (M^+^) calcd for C_16_H_12_O_4_: 268.0736, Found: 268.0734.

#### 3.2.8. Methyl 2-((3-Methoxyphenyl)thio)acetate (**9**)

Methyl bromoacetate (0.18 mL, 1.95 mmol) was added to a stirred suspension of compound **8** (0.228 g, 1.63 mmol) and K_2_CO_3_ (0.67 g, 4.88 mmol) in acetone (6 mL) under an argon atmosphere at room temperature. After 5 h of refluxing, the reaction mixture was brought to room temperature, filtered through Celite^®^, and washed with acetone (10 mL). Then, the filtrate was concentrated in vacuo. The residue was dissolved in EtOAc (50 mL), washed with 1N HCl (10 mL), distilled H_2_O (2 × 15 mL) and brine (15 mL), dried over anhydrous Na_2_SO_4_, and concentrated in vacuo. The residue was purified by column chromatography (EtOAc/hexane = 1/8) to afford compound **9** (0.26 g, 74.3%) as a clear liquid. ^1^H NMR (300 MHz, CDCl_3_) δ: 3.65 (s, 2H), 3.71 (s, 3H), 3.78 (s, 3H), 6.74 (d, *J* = 7.2 Hz, 1H), 6.93 (s, 1H), 6.94 (d, *J* = 7.8 Hz, 1H), 7.19 (t, *J* = 7.9 Hz, 1H); ^13^C NMR (75 MHz, CDCl_3_) δ: 36.5, 52.5, 55,3, 112.9, 115.1, 121.8, 130.0, 136.3, 159.9, 169.8; GC-MS *m*/*z* 212 (M^+^, base).

#### 3.2.9. Methyl 3-(Dimethylamino)-2-((3-methoxyphenyl)thio)acrylate (**10**)

A solution of compound **9** (9.0 g, 42.40 mmol) in *N*,*N*-dimethylformamide dimethyl acetal (6.1 mL, 45.93 mmol) was stirred for 10 h at 80 °C under argon. After the completion of the reaction, the reaction mixture was brought to room temperature. The residue resulting from in vacuo solvent evaporation was purified by column chromatography (EtOAc/hexane = 1/4) to obtain compound **10** (11.31 g, 99.8%) as a brown liquid. ^1^H NMR (300 MHz, CDCl_3_) δ: 3.08 (s, 6H), 3.57 (s, 3H), 3.65 (s, 3H), 6.50 (d, *J* = 7.8 Hz, 1H), 6.60 (s, 1H), 6.63 (d, *J* = 8.1 Hz, 1H), 7.04 (t, *J* = 7.8 Hz, 1H) 7.98 (s, 1H); ^13^C NMR (75 MHz, CDCl_3_) δ: 42.6, 51.3, 54.8, 81.7, 109.6, 110.2, 116.8, 129.1, 129.3, 142.2, 155.3, 159.7, 169.6; GC-MS *m*/*z* 267 (M^+^, base).

#### 3.2.10. Methyl 6-Methoxybenzo[*b*]thiophene-2-carboxylate (**11**)

A mixture of compound **10** (1.00 g, 3.74 mmol) and iodine (2.56 g, 10.10 mmol) in anhydrous CH_2_Cl_2_ (10 mL) was refluxed for 12 h under argon. After the completion of the reaction, the reaction mixture was diluted with CH_2_Cl_2_ (30 mL), washed sequentially with aq. sat. Na_2_S_2_O_3_ (2 × 15 mL), distilled H_2_O (2 × 15 mL) and brine (15 mL), dried over anhydrous Na_2_SO_4_, and concentrated in vacuo. The residue was purified with column chromatography (EtOAc/hexane = 1/30) to afford compound **11** (0.26 g, 1.17 mmol, 31.3%) as a white solid. ^1^H NMR (300 MHz, CDCl_3_) δ 3.88 (s, 3H), 3.92 (s, 3H), 7.00 (dd, *J* = 8.8, 2.0 Hz, 1H), 7.26 (s, 1H), 7.72 (d, *J* = 8.8 Hz, 1H), 7.95 (s, 1H); ^13^C NMR (75 MHz, CDCl_3_) δ: 52.2, 55.7, 104.6, 115.7, 126.2, 130.3, 130.7, 132.8, 144.3, 159.5, 163.1; GC-MS *m*/*z* 222 (M^+^, base).

#### 3.2.11. Methyl 6-Hydroxybenzo[*b*]thiophene-2-carboxylate (**11.1**)

BBr_3_ (1.0 M solution in CH_2_Cl_2_, 2.2 mL, 2.24 mmol) was added slowly to a stirred solution of compound **11** (0.25 g, 1.12 mmol) in anhydrous CH_2_Cl_2_ (5 mL) at 0 °C under an argon atmosphere. After stirring for 2 h at 0 °C and an additional 2 h at room temperature, the resulting mixture was cooled again to 0 °C. Then, MeOH (2 mL) was added slowly to quench the excess BBr_3_, followed by 20 min of stirring at room temperature. The reaction mixture was diluted with CH_2_Cl_2_ (35 mL), washed with distilled H_2_O (2 × 10 mL) and brine (10 mL), dried over anhydrous Na_2_SO_4_, and concentrated in vacuo. The residue was purified by column chromatography (EtOAc/hexane = 1/8) to obtain compound **11.1** (0.19 g, 82.6 %) as a white solid. ^1^H NMR (300 MHz, CD_3_OD) δ: 3.87 (s, 3H), 6.92 (d, *J* = 8.8 Hz, 1H), 7.20 (s, 1H), 7.71 (d, *J* = 8.8 Hz, 1H) 7.92 (s, 1H); ^13^C NMR (75 MHz, CD_3_OD) δ: 52.7, 107.8, 116.8, 127.5, 130.5, 131.7, 133.2, 145.6, 158.8, 164.8; GC-MS *m*/*z* 208 (M^+^, base).

#### 3.2.12. Methyl 6-(Benzyloxy)benzo[*b*]thiophene-2-carboxylate (**11.2**)

Benzyl bromide (0.57 mL, 4.80 mmol) was added to a suspension of compound **11.1** (1.00 g, 4.80 mmol) and K_2_CO_3_ (1.33 g, 9.60 mmol) in acetone (20 mL) under an argon atmosphere at room temperature. After 10 h of refluxing, the reaction mixture was brought to room temperature, filtered through Celite^®^, and washed with acetone (10 mL). The filtrate was concentrated in vacuo. The residue was dissolved in EtOAc (45 mL), washed with 1N HCl (10 mL), distilled H_2_O (2 × 10 mL) and brine (10 mL), dried over anhydrous Na_2_SO_4_, and concentrated under reduced pressure. The residue was purified by column chromatography (EtOAhc/hexane = 1/10) to obtain compound **11.2** (1.42 g, 4.76 mmol, 99.3%) as a white solid. ^1^H NMR (300 MHz, CDCl_3_) δ: 3.91 (s, 3H), 5.12, (s, 2H), 7.08 (dd, *J* = 8.8, 1.9 Hz, 1H), 7.34–7.45 (m, 6H), 7.73 (d, *J* = 8.8 Hz, 1H), 7.96 (s, 1H); ^13^C NMR (75 MHz, CDCl_3_) δ: 52.3, 70.6, 105.9, 116.2, 126.3, 127.4, 128.1, 128.6, 130.3, 130.9, 133.0, 136.5, 144.1, 158.6, 163.1; GC-MS *m*/*z* 298 (M^+^, base).

#### 3.2.13. (6-(Benzyloxy)benzo[*b*]thiophen-2-yl)methanol (**11.3**)

Following the same method used for the preparation of compound **7.3**, treatment of compound **11.2** (1.4 g, 4.69 mmol) in anhydrous THF (10 mL) with LiAlH_4_ (2.0 M in THF, 5.6 mL, 5.63 mmol, 1.2 eq.) afforded compound **11.3** (1.26 g, 99.2%) as a white solid. ^1^H NMR (300 MHz, CDCl_3_) δ: 2.13 (br s, 1H), 4.83 (s, 2H), 5.08 (s, 2H), 7.02 (dd, *J* = 8.8, 2.0 Hz, 1H), 7.01 (s, 1H), 7.31–7.44 (m, 6H), 7.57 (d, *J* = 8.8 Hz, 1H); ^13^C NMR (75 MHz, CDCl_3_) δ: 60.9, 70.7, 106.8, 115.0, 121.1, 124.1, 127.4, 127.9, 128.5, 133.8, 137.0, 141.4, 142.3, 156.6; GC-MS *m*/*z* 270 (M^+^, 100%).

#### 3.2.14. 6-(Benzyloxy)benzo[*b*]thiophene-2-carbaldehyde (**11.4**)

Following the same method used for the preparation of compound **7.4**, treatment of compound **11.3** (1.26 g, 4.66 mmol) with IBX (1.96 g, 6.99 mmol) in DMSO (20 mL) afforded **11.4** (1.18 g, 94.4%) as a yellow solid. ^1^H NMR (300 MHz, CDCl_3_) δ: 5.15 (s, 2H), 7.11 (dd, *J* = 8.8, 2.4 Hz, 1H), 7.34–7.45 (m, 6H), 7.80 (d, *J* = 8.8 Hz, 1H), 7.92 (s, 1H), 9.99 (s, 1H); ^13^C NMR (75 MHz, CDCl_3_) δ: 70.9, 106.5, 117.0, 127.2, 127.6, 128.3, 128.8, 139.1, 134.2, 136.5, 141.6, 145.2, 159.7, 183.8; GC-MS *m*/*z* 268 ([M]^+^, 100%).

#### 3.2.15. (*E*)-6-(Benzyloxy)-2-(3,5-bis(benzyloxy)styryl)benzo[*b*]thiophene (**11.5**)

Following the same method used for the preparation of compound **7.5**, treatment of diethyl 3,5-bis(benzyloxy)benzylphosphonate (1.94 g, 4.40 mmol) in DMF (6 mL) with NaH (60% in mineral oil, 0.32 g, 13.19 mmol) in DMF (4 mL), followed by the addition of compound **11.4** (1.18 g, 4.40 mmol) in DMF (6 mL), afforded compound **11.5** (1.99 g, 81.6%) as a yellow solid. ^1^H NMR (300 MHz, CDCl_3_) δ: 5.03 (s, 4H), 5.70 (s, 2H), 6.53 (s, 1H), 6.71 (s, 2H), 6.78 (d, *J* = 16.1 Hz, 1H), 6.98 (d, *J* = 8.8 Hz, 1H), 7.11 (s, 1H), 7.20 (d, *J* = 16.1 Hz, 1H), 7.28–7.40 (m, 16H), 7.53 (d, *J* = 8.8 Hz, 1H); ^13^C NMR (75 MHz, CDCl_3_) δ: 70.4, 70.7, 102.1, 102.2, 106.1, 106.3, 106.7, 115.0, 123.0, 124.0, 127.4, 127.9, 128.5, 129.3, 129.7, 134.5, 136.9, 137.0, 138.9, 139.3, 140.5, 140.6, 157.1, 160.2; MS (EI^+^) *m*/*z* 554 (M^+^, base), 463, 281, 91.

#### 3.2.16. (*E*)-5-(2-(6-Hydroxybenzo[*b*]thiophen-2-yl)vinyl)benzene-1,3-diol (**4**)

Following the same method used for the preparation of compound **3**, treatment of compound **11.5** (0.16 g, 0.29 mmol) and *N*,*N*-dimethylaniline (0.44 mL, 3.47 mmol) in anhydrous CH_2_Cl_2_ (10 mL) with AlCl_3_ (0.61 g, 4.61 mmol) afforded compound **4** (0.03 g, 37.5 %) as a yellow solid. ^1^H NMR (300 MHz, (CD_3_)_2_CO) δ: 6.31 (s, 1H), 6.56, (s, 2H), 6.75 (d, *J* = 16.1 Hz, 1H), 6.90 (d, *J* = 8.4 Hz, 1H), 7.25 (s, 1H), 7.27 (s, 1H) 7.33 (d, *J* = 16.1 Hz, 1H), 7.58 (d, *J* = 8.4 Hz, 1H) 8.41 (br s, 3H); ^13^C NMR (75 MHz, (CD_3_)_2_CO) δ: 103.6, 106.1, 108.3, 115.5, 123.4, 124.2, 125.1, 130.4, 134.6, 139.9, 140.5, 141.5, 156.6, 159.6; MS (EI^+^) *m*/*z* 284 (M^+^, base) HRMS *m*/*z* (M^+^) calcd for C_16_H_12_O_3_S: 284.0507, Found: 284.0510.

#### 3.2.17. 4-Methoxy-2-(methylselanyl)benzaldehyde (**13**)

A mixture of dimethyl diselenide (1.99 g, 10.57 mmol) and dithiothreitol (0.98 g, 6.34 mmol) in anhydrous DMF (8 mL) was stirred for 1 h at 80 °C under an argon atmosphere. To this mixture, compound **12** (1.63 g, 10.57 mmol) and DBU (3.95 mL, 26.42 mmol) were added. The resulting mixture was heated with stirring for another 12 h. After the reaction was completed, the reaction mixture was brought to room temperature, and distilled H_2_O (10 mL) was added to it. After extraction with EtOAc (3 × 30 mL), the organic layer was washed with distilled H_2_O (3 × 15 mL) and brine (2 × 15 mL), dried over anhydrous Na_2_SO_4_, and concentrated in vacuo. The residue was purified by column chromatography (EtOAc/hexane = 1/8) to obtain compound **13** (1.80 g, 74.4%) as a yellow solid. ^1^H NMR (300 MHz, CDCl_3_) δ: 2.26 (s, 3H), 3.90 (s, 3H), 6.81 (dd, *J* = 8.4, 2.2 Hz, 1H), 6.90 (s, 1H), 7.71 (d, *J* = 8.4 Hz, 1H), 9.95 (s, 1H); ^13^C NMR (75 MHz, CDCl_3_) δ: 5.8, 55.6, 110.1, 114.2, 128.5, 136.9, 140.8, 163.8, 190,3; MS (EI^+^) *m*/*z* 230 (M^+^), 215 (base), 187, 135.

#### 3.2.18. Methyl 2-((2-Formyl-5-methoxyphenyl)selanyl)acetate (**14**)

A mixture of compound **13** (1.30 g, 5.67 mmol) and methyl bromoacetate (10.10 mL, 113.47 mmol) was refluxed for 3 h under an argon atmosphere. After the reaction was completed, the reaction mixture was brought to room temperature, and the evaporation of methyl bromoacetate yielded compound **14** (1.63 g, quantitative) as a white solid. ^1^H NMR (300 MHz, CDCl_3_) δ: 3.56 (s, 2H), 3.72 (s, 3H), 3.93 (s, 3H), 6.85 (dd, *J* = 8.7, 1.8 Hz, 1H), 7.29 (s, 1H), 7.72 (d, *J* = 8.7 Hz, 1H) 9.92 (s, 1H); ^13^C NMR (75 MHz, CDCl_3_) δ: 25.2, 52.5, 55.8, 111.9, 114.6, 128.2, 136.7, 139.2, 164.0, 171.1, 190.4; MS (EI^+^) *m*/*z* 288 (M^+^), 286, 214 (base), 187.

#### 3.2.19. Methyl 6-Methoxybenzo[*b*]selenophene-2-carboxylate (**15**)

A suspension of compound **14** (0.48 g, 1.67 mmol) and K_2_CO_3_ (0.69 g, 5.01 mmol) in anhydrous CH_3_CN (15 mL) was refluxed for 4 h under an argon atmosphere. After the reaction was completed, the reaction mixture was brought to room temperature, filtered through a Celite^®^ pad, and washed with CH_3_CN (10 mL). The filtrate was concentrated under reduced pressure. The residue was dissolved in EtOAc (45 mL), washed sequentially with 1N HCl (9 mL), H_2_O (2 × 10 mL) and brine (10 mL), dried over anhydrous Na_2_SO_4_ and concentrated under reduced pressure to yield compound **15** (0.42 g, 93.3%) as a white solid. ^1^H NMR (300 MHz, CDCl_3_) δ: 3.86 (s, 3H), 3.89 (s, 3H), 6.98 (dd, *J* = 8.7, 2.4 Hz, 1H), 7.34 (s, 1H), 7.73 (d, *J* = 8.7 Hz, 1H), 8.18 (s, 1H); ^13^C NMR (75 MHz, CDCl_3_) δ: 52.3, 55.6, 108.4, 115.0, 127.9, 132.7, 134.1, 135.0, 145.9, 159.3, 164.1; MS (EI^+^) *m*/*z* 270 (M^+^, base), 268, 239, 168.

#### 3.2.20. Methyl 6-Hydroxybenzo[*b*]selenophene-2-carboxylate (**15.1**)

Following the method used to prepare compound **11.1**, treatment of compound **15** (0.22 g, 0.82 mmol) in CH_2_Cl_2_ (10 mL) with BBr_3_ (1.0 M in CH_2_Cl_2;_ 0.32 mL, 3.44 mmol) afforded compound **15.1** (0.20 g, 93.3%) as a white solid. ^1^H NMR (300 MHz, DMSO-*d*_6_) δ: 3.82 (s, 3H), 6.91 (dd, *J* = 8.4, 1.2 Hz, 1H), 7.44 (s, 1H), 7.82 (d, *J* = 8.4 Hz, 1H), 8.24 (s, 1H), 10.11 (br s, 1H); ^13^C NMR (75 MHz, DMSO-*d*_6_) δ: 62.0, 120.8, 125.2, 138.2, 140.3, 143.2, 144.2, 154.9, 167.1, 173.3; MS (EI^+^) *m*/*z* 256 (M^+^, base), 254, 225, 197.

#### 3.2.21. Methyl 6-(Benzyloxy)benzo[*b*]selenophene-2-carboxylate (**15.2**)

Following the method used to prepare compound **7.2**, treatment of compound **15.1** (0.19 g, 0.74 mmol) and K_2_CO_3_ (0.21 g, 1.49 mmol) in acetone (10 mL) with benzyl bromide (0.10 mL, 0.82 mmol) yielded compound **15.2** (0.26 g, quantitative) as a white solid. ^1^H NMR (300 MHz, CDCl_3_) δ: 3.89 (s, 3H), 5.11 (s, 2H), 7.06 (dd, *J* = 8.7, 2.3 Hz, 1H), 7.33–7.44 (m, 6H), 7.75 (d, *J* = 8.7 Hz, 1H), 8.18 (s, 1H); ^13^C NMR (75 MHz, CDCl_3_) δ:52.4, 70.6, 109.8, 115.7, 127.3, 128.1, 128.6, 133.0, 134.1, 135.3, 136.5, 145.9, 158.5, 164.2; MS (EI^+^) *m*/*z* 346 (M^+^, base), 344, 255, 168.

#### 3.2.22. (6-(Benzyloxy)benzo[*b*]selenophen-2-yl)methanol (**15.3**)

Following the method used to prepare compound **7.3**, treatment of compound **15.2** (1.1 g, 3.19 mmol) in THF (20 mL) with LiAlH_4_ (2.0 M in THF, 1.91 mL, 3.82 mmol) yielded compound **15.3** (1.01 g, 99.0%) as a white solid. ^1^H NMR (300 MHz, CDCl_3_) δ: 2.15 (br s, 1H), 4.85 (d, *J* = 5.4 Hz, 2H), 5.08 (s, 2H), 7.00 (d, *J* = 8.4 Hz, 1H), 7.24 (s, 1H), 7.34–7.44 (m, 6H), 7.56 (d, *J* = 8.4 Hz, 1H); ^13^C NMR (75 MHz, CDCl_3_) δ: 62.9, 70.7, 110.5, 114.6, 124.0, 125.6, 127.4, 127.9, 128.5, 136.0, 137.0, 142.6, 146.4, 156.5; MS (EI^+^) *m*/*z* 318 (M^+^, base), 316, 227, 91.

#### 3.2.23. 6-(Benzyloxy)benzo[*b*]selenophene-2-carbaldehyde (**15.4**)

Following the method used to prepare compound **7.4**, treatment of compound **15.3** (0.21 g, 0.66 mmol) with IBX (0.28 g, 0.99 mmol) in DMSO (3 mL) yielded compound **15.4** (0.21 g, quantitative) as a yellow solid. ^1^H NMR (300 MHz, CDCl_3_) δ: 5.14 (s, 2H), 7.09 (dd, *J* = 8.7, 2.1 Hz, 1H), 7.34–7.45 (m, 6H), 7.82 (d, *J* = 8.7 Hz, 1H), 8.15 (s, 1H), 9.89 (s, 1H); ^13^C NMR (75 MHz, CDCl_3_) δ: 70.7, 110.2, 116.1, 127.4, 128.2, 128.6, 128.8, 135.1, 136.3, 138.1, 144.5, 146.1, 159.3, 184.9; LRMS (EI^+^) *m*/*z* 316 (M^+^, base), 225, 169, 167.

#### 3.2.24. (*E*)-6-(Benzyloxy)-2-(3,5-bis(benzyloxy)styryl)benzo[*b*]selenophene (**15.5**)

Following the method used to prepare compound **7.5**, treatment of diethyl 3,5-bis(benzyloxy)benzylphosphonate (0.28 g, 0.63 mmol) in DMF (6 mL) with NaH (60% in mineral oil, 0.046 g, 1.90 mmol) in DMF (4 mL), followed by the addition of compound **15.4** (0.2 g, 0.63 mmol) in DMF (6 mL), afforded compound **15.5** (0.32g, 84.2%) as a yellow solid. ^1^H NMR (300 MHz, CDCl_3_) δ: 5.04 (s, 4H), 5.09 (s, 2H), 6.53 (s, 1H), 6.62 (d, *J* = 15.9 Hz, 1H) 6.71 (s, 2H) 6.97 (d, *J* = 9.3 Hz, 1H), 7.22 (d, *J* = 15.9 Hz, 1H), 7.29–7.40 (m, 17H), 7.54 (d, *J* = 8.7 Hz, 1H); ^13^C NMR (75 MHz, CDCl_3_) δ: 70.4, 70.7, 102.3, 106.1, 110.5, 114.7, 125.3, 125.6, 126.9, 127.3, 127.4, 127.9, 128.5, 130.9, 136.8, 136.9, 137.0, 139.0, 141.2, 143.3, 157.1, 160.3; MS (EI^+^) *m*/*z* 602 (M^+^, base), 600, 603, 329.

#### 3.2.25. (*E*)-5-(2-(6-Hydroxybenzo[*b*]selenophen-2-yl)vinyl)benzene-1,3-diol (**5**)

Following the method used to prepare compound **3**, treatment of compound **15.5** (0.27 g, 0.45 mmol) and *N*,*N*-dimethylaniline (1.02 mL, 8.08 mmol) in anhydrous CH_2_Cl_2_ (10 mL) with AlCl_3_ (1.44 g, 10.77 mmol) afforded compound **5** (0.06 g, 40.0%) as a yellow solid. ^1^H NMR (300 MHz, (CD_3_)_2_CO) δ: 6.30 (t, *J* = 1.5 Hz, 1H), 6.55, (d, *J* = 1.5 Hz, 2H), 6.61 (d, *J* = 15.6 Hz, 1H), 6.87 (dd, *J* = 8.4, 1.8 Hz, 1H), 7.32 (d, *J* = 15.6 Hz, 1H), 7.35 (d, *J* = 1.8 Hz, 1H), 7.40 (s, 1H), 7.56 (d, *J* = 8.4 Hz, 1H), 8.34 (br s, 3H); ^13^C NMR (75 MHz, (CD_3_)_2_CO) δ: 103.6, 106.1, 112.1, 115.3, 125.7, 126.6, 128.0, 131.6, 136.8, 140.0, 142.0, 143.2, 156.6, 159.6; MS (EI^+^) *m*/*z* 332 (M^+^, base), 330, 252; HRMS *m*/*z* (M^+^) calcd for C_16_H_12_O_3_Se: 331.9952, Found: 331.9954.

### 3.3. Measurement of Antioxidant Activity Using ABTS

The radical cation solution was freshly produced by blending equal volumes of 7 mM ABTS (2,2′-azino-bis(3-ethylbenzothiazoline-6-sulfonic acid)) stock solution and 2.45 mM potassium persulfate stock solution. This mixture was kept at 0 °C in the dark for at least 12 h. This solution was then diluted with methanol to achieve a UV absorption value of 1.00 at 734 nm. Compounds **1**–**5** were also dissolved in methanol to make 1000 µM stock solutions, which were subsequently diluted with methanol to final concentrations of 7.80, 15.6, 31.25, 62.5, 125, 250, 500, and 1000 µM. In three separate sets of test tubes, 0.9 mL of the ABTS solution was mixed with 0.1 mL of each compound’s solution in the dark. After 30 min of incubation, UV absorption at 734 nm was recorded. A mixture with 0.9 mL of ABTS and 0.1 mL of methanol was used as a control solution. UV-1800 (Shimadzu Corporation, Kyoto, Japan) was used to measure UV absorption. Radical scavenging rates were determined from the UV absorption data, and the IC_50_ values were computed with Origin 8 software (OriginLab Corporation, Northampton, MA, USA).

### 3.4. Computational Method

Geometric optimizations and energy calculations for all compounds were conducted in the aqueous phase at the B3LYP/6-31+G(d,p) level of theory. Frequency calculations at the same level of theory were performed to determine the optimized geometries with no imaginary frequency. The MeOH solvent effect was included using an integral equation formalism of the polarizable continuum model (IEFPCM). All calculations were carried out using Gaussian 09 programs [22].

## 4. Conclusions

Aromaticity-extended resveratrol derivatives **3**, **4**, and **5**, incorporating benzofuran, benzothiophene, and benzoselenophene, respectively, were synthesized via ring closure and Horner–Wadsworth–Emmons reactions. The antioxidant activities of the synthesized derivatives were assessed using an ABST assay. Compared to the parent, resveratrol **1**, resveratrol derivatives **3**–**5** exhibited higher radical scavenging activities, with IC_50_ values in the range of 2.75–3.39 μM. The presence of aromaticity-extended benzoheterols in resveratrol derivatives **3**–**5** facilitated the stabilization of the radical species via delocalization of the π electrons, thereby improving the antioxidant activities of the systems. In addition, derivatives **3**, **4**, and **5** fused with heterocyclic compounds exhibited improved ABTS radical scavenging activity compared to resveratrol analogue **2** with a naphthalene moiety. B3LYP calculations confirmed the efficient π extension in compounds **3**–**5** based on the decreased HOMO–LUMO gaps compared to those of resveratrol **1** and derivative **2**. Therefore, aromaticity extension by incorporation of benzofuran, benzothiophene, and benzoselenophene presents a viable approach for synthesizing efficient antioxidant agents. The novel resveratrol derivatives with fused heterocyclic rings have the potential to enhance the health benefits of resveratrol and offer new avenues for the development of advanced functional materials and therapeutic agents.

## Figures and Tables

**Table 1:** IC_50_ values of resveratrol **1** and aromaticity-extended resveratrol derivatives (**2**–**5**) in ABTS assay.

Compound	IC_50_ (μM)
**1**	5.15 ± 0.35
**2**	4.16 ± 0.12
**3**	3.39 ± 0.59
**4**	3.02 ± 0.30
**5**	2.75 ± 0.30

**Table 2:** Calculated HOMO–LUMO energy levels and energy gaps of aromaticity-extended resveratrol derivatives (**2**–**5**) in the gas phase and MeOH at the B3LYP/6-31G+(d,p) level of theory.

Compound	Gas Phase			MeOH		
	HOMO (eV)	LUMO (eV)	ΔE_H-L_ (eV)	HOMO (eV)	LUMO (eV)	ΔE_H-L_ (eV)
**1**	−5.58	−1.64	3.94	−5.66	−1.72	3.94
**2**	−5.55	−1.78	3.77	−5.61	−1.86	3.76
**3**	−5.42	−1.89	3.53	−5.51	−1.99	3.52
**4**	−5.49	−1.94	3.55	−5.56	−2.01	3.55
**5**	−5.50	−1.97	3.53	−5.56	−2.03	3.53

## Data Availability

The data presented in this study are available upon request.

## Supplementary Materials

The supporting information can be downloaded at: https://www.mdpi.com/article/10.3390/ijms26125872/s1.
