# Supplementary material for: Design, Synthesis, Theoretical Study, and Antioxidant Activity of Aromaticity-Extended Resveratrol Derivatives Incorporating Chalcogen
Source: Int J Mol Sci. 2025 Jun 19;26(12):5872. doi: 10.3390/ijms26125872 (PMC12193106; doi:10.3390/ijms26125872)
Supplement: Supplementary file 1 [file ijms-26-05872-s001.zip › ijms-3675486-supplementary.pdf]

Supporting Information for

Design, synthesis, theoretical study, and antioxidant activity of aromaticity-extended resveratrol derivatives incorporating chalcogen

Sangwon Ko [1,†], Hyun Min Lim [2,†], Yeonho Song [3], Hyonseok Hwang [3,*] and Jeong Tae Lee [2,*]

[1] Transportation Environmental Research Department, Korea Railroad Research Institute, Uiwang 16105, Korea

[2] Department of Chemistry and Institute of Applied Chemistry, Hallym University, Chuncheon 24252, Korea

[3] Department of Chemistry and Institute for Molecular Science and Fusion Technology, Kangwon National University, Chuncheon 24341, Korea

* Correspondence: hhwang@kangwon.ac.kr (H. H.), leo900516@gmail.com and jtshl@hallym.ac.kr (J.T.L.)

[†] These authors contributed equally to this work.

HRMS, ^{1}H-, and ^{13}C-NMR spectra of compound 3, 4, and 5

[Mass Spectrum]
Data : benzofuran type resveratrol HR   Date : 22-Sep-2016 15:40
RT : 1.03 min   Scan# : 29
Elements : C 16/0, H 34/0, O 4/0
Mass Tolerance   : 1000ppm, 5mmu if m/z < 5, 50mmu if m/z > 50
Unsaturation (U.S.) : -0.5 - 20.0

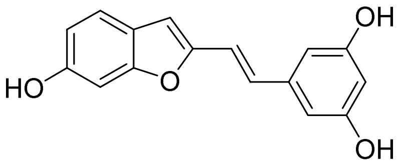

3

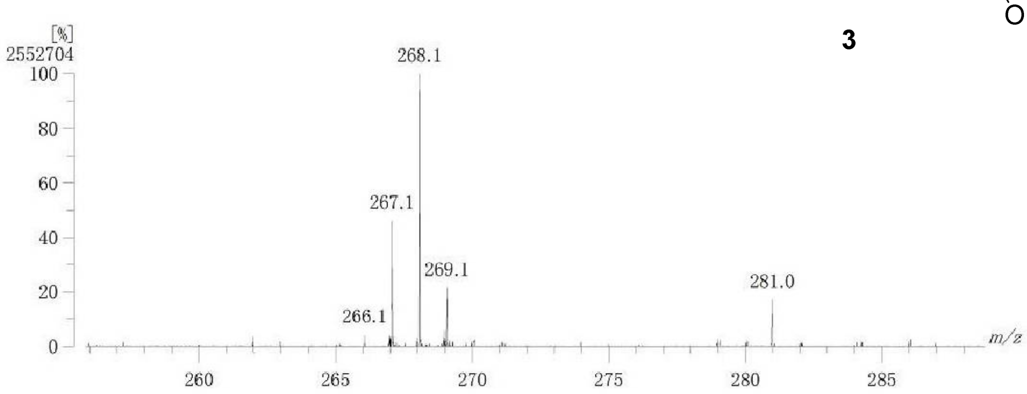

	Observed m/z	Int%	Err[ppm / mmu]	U.S.	Composition
1	268.0734	100.00	-0.6 / -0.2	11.0	C16 H12 O4

HRMS spectra of compound 3

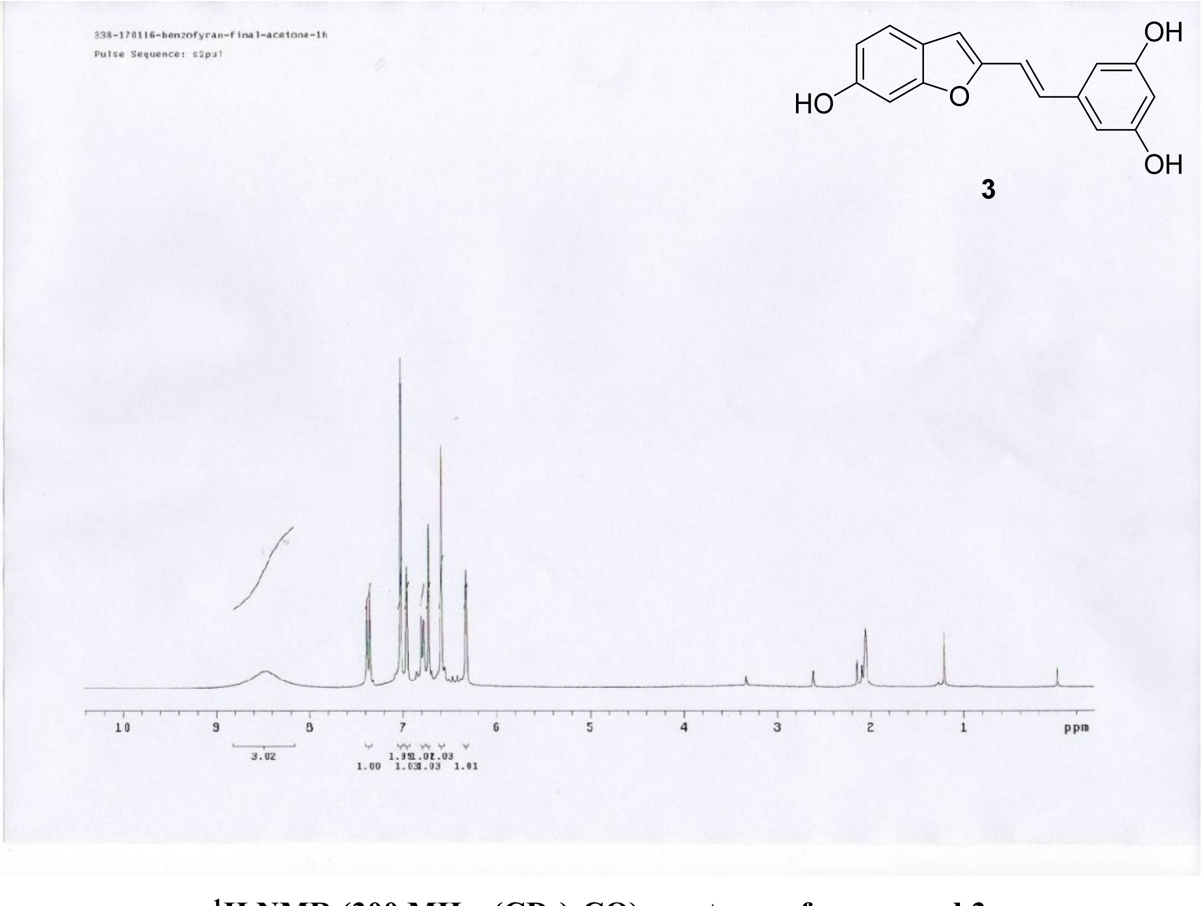

^{1}H NMR (300 MHz, $(CD_3)_2CO$) spectrum of compound 3

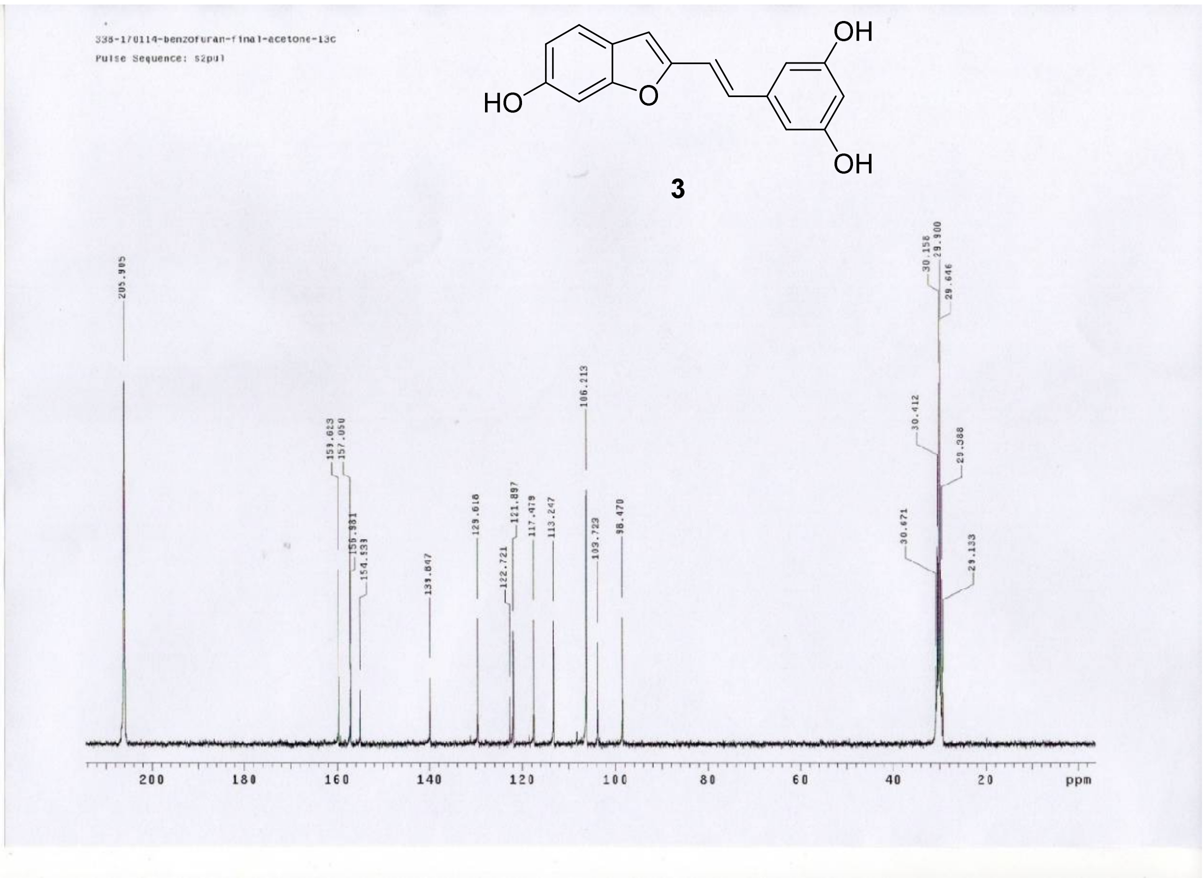

^{13}C NMR (75 MHz, $(CD_3)_2CO$) spectrum of compound 3

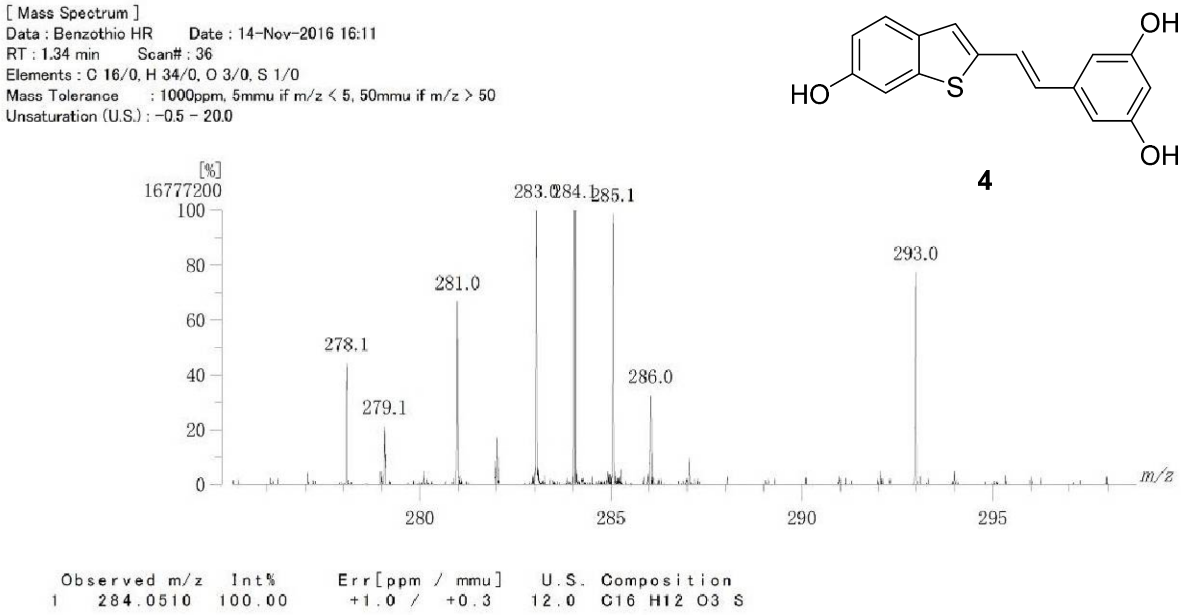

HRMS spectra of compound 4

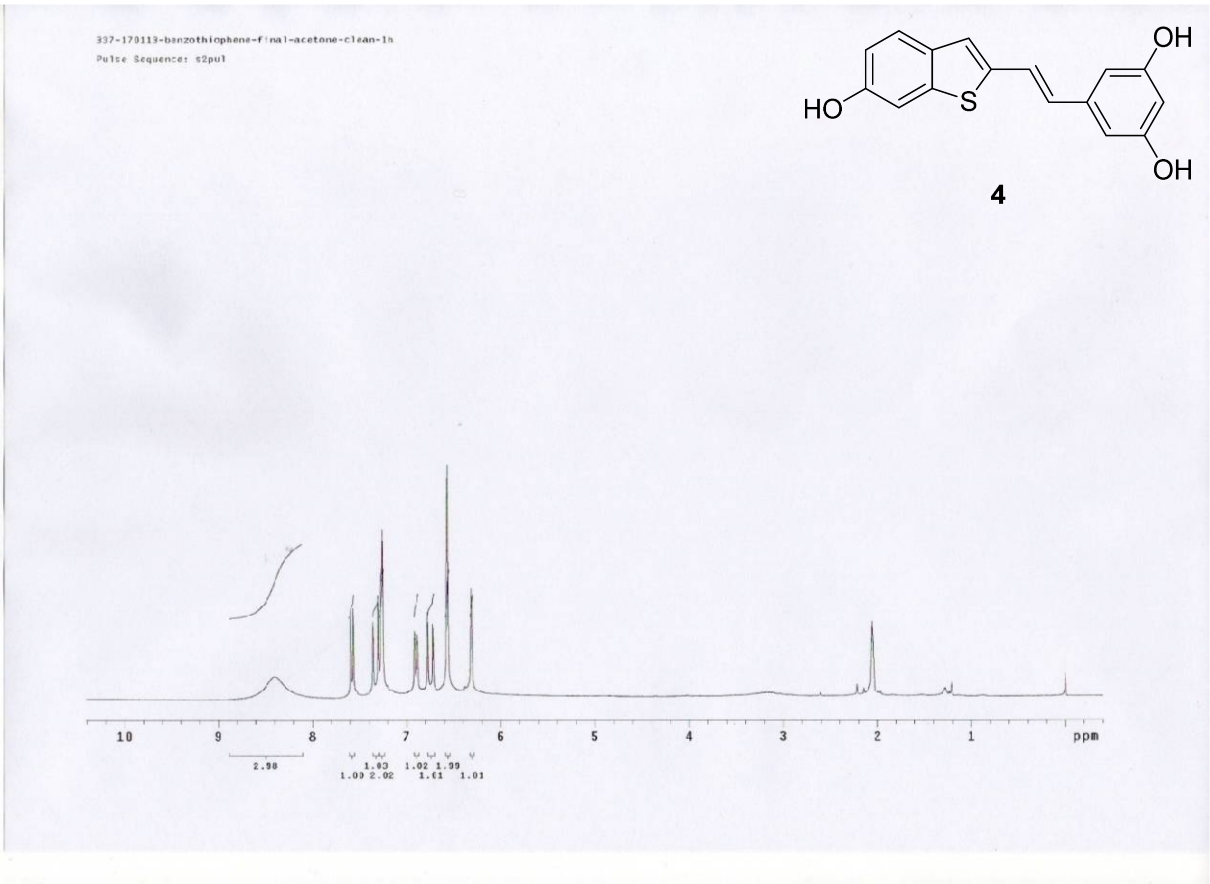

^{1}H NMR (300 MHz, $(CD_3)_2CO$) spectrum of compound 4

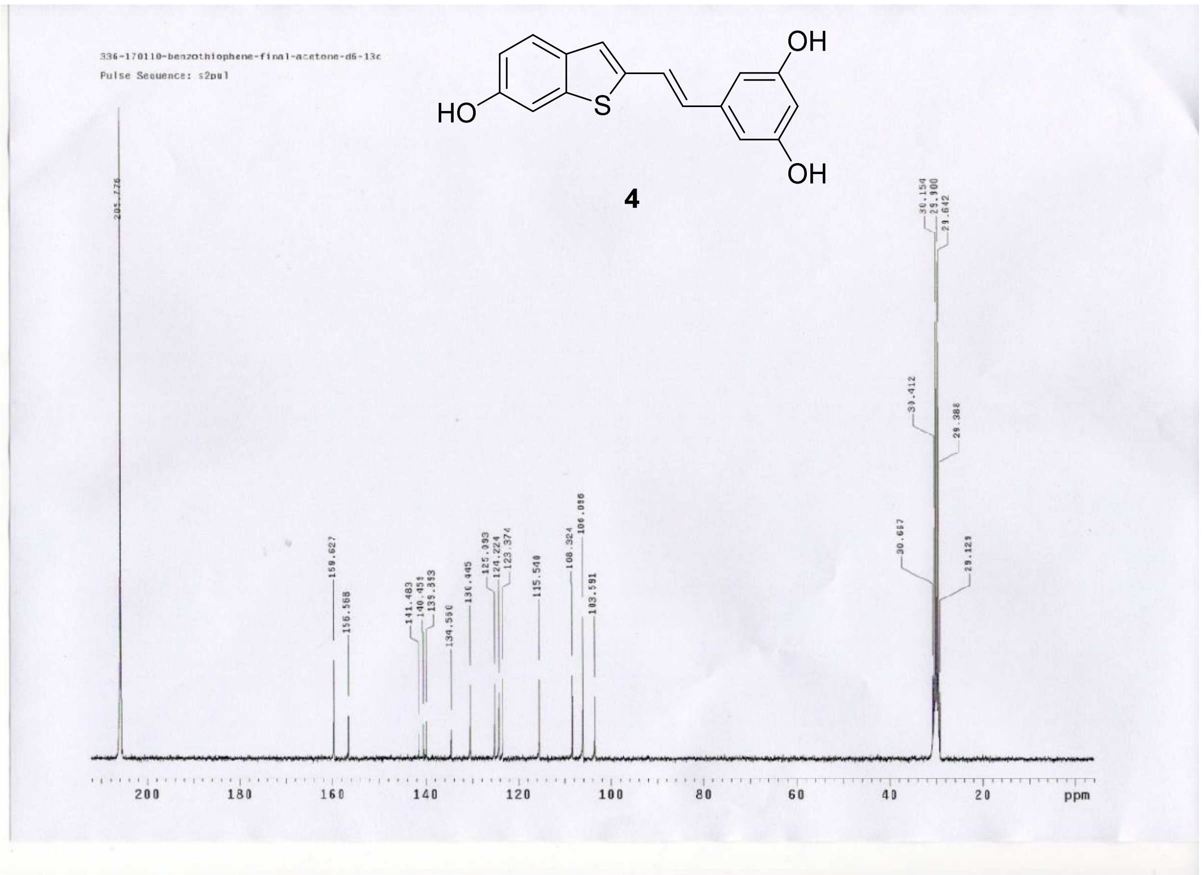

^{13}C NMR (75 MHz, $(CD_3)_2CO$) spectrum of compound 4

[Mass Spectrum]
Data : sample 7 HR   Date : 12-Jan-2017 15:56
RT : 2.07 min   Scan# : 55
Elements : C 16/0, H 34/0, O 3/0, 78Se 1/0, 80Se 1/0
Mass Tolerance   : 5mmu
Unsaturation (U.S.) : 10.0 - 20.0

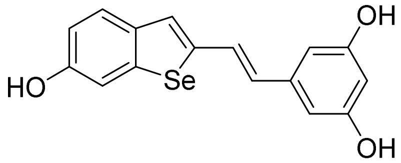

5

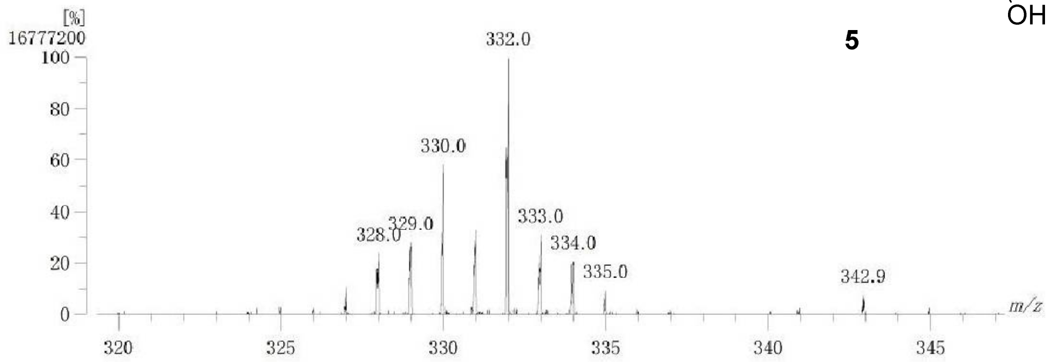

	Observed m/z	Int%	Err[ppm / mmu]	U.S.	Composition
1	331.9954	100.00	+0.7 / +0.2	12.0	C16 H12 O3 80Se

HRMS spectra of compound 5

361-170224-benzoseleno-final-acetone-1h
Pulse Sequence: s2pul

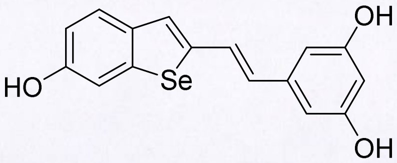

5

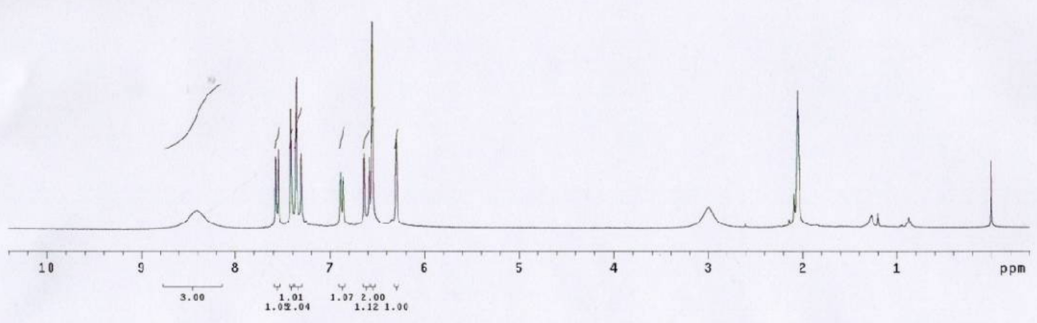

^{1}H NMR (300 MHz, $(CD_3)_2CO$) spectrum of compound 5

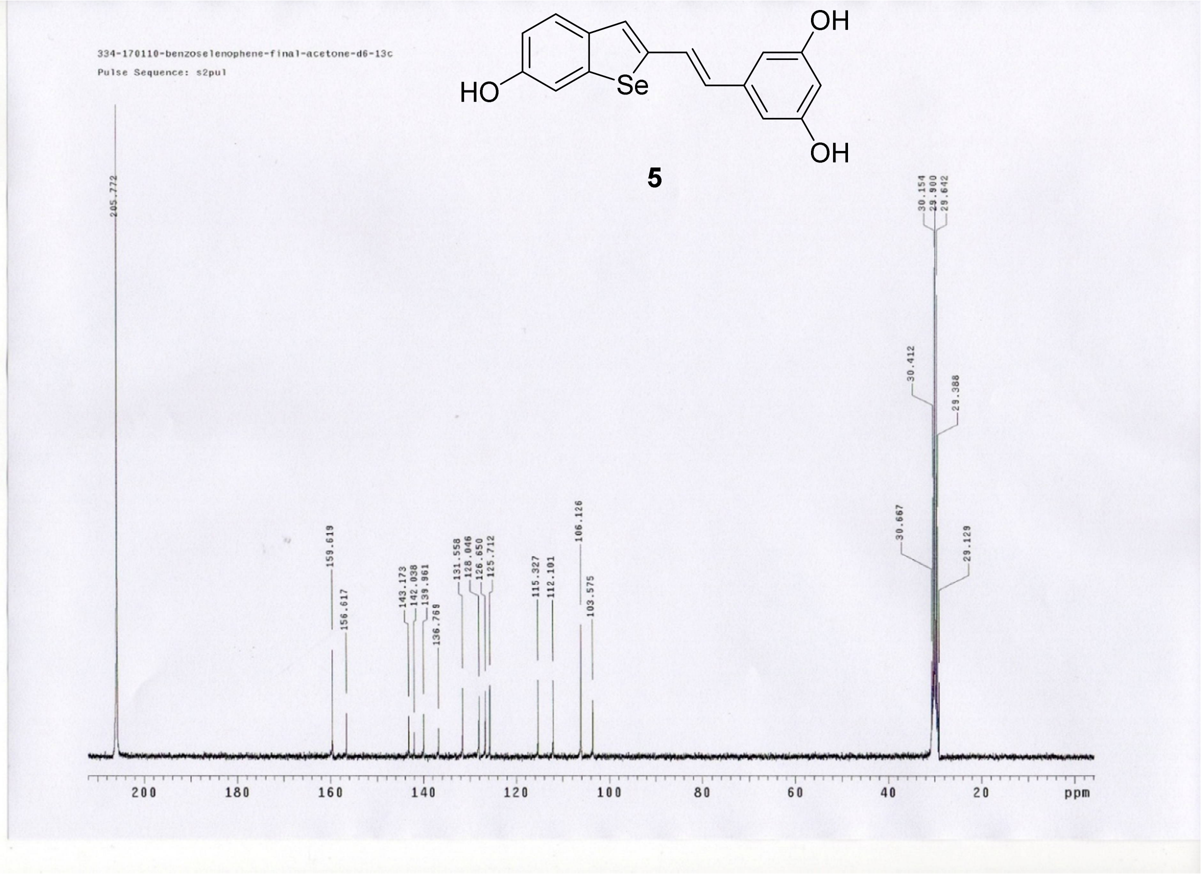

^{13}C NMR (75 MHz, $(CD_3)_2CO$) spectrum of compound 5

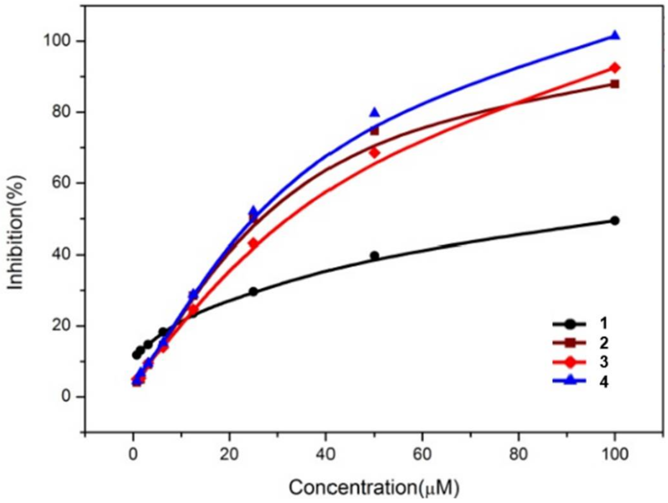

Figure S1. Radical scavenging activities of resveratrol **1** and aromaticity extended resveratrol derivatives (**3**–**5**) in DPPH assay.

Table S1. IC_{50} values of resveratrol **1** and aromaticity-extended resveratrol derivatives (**3**–**5**) in ABTS assay.

Compound	IC_{50} (μM)
1	> 100
3	40.20±0.89
4	46.52±3.42
5	36.71±1.41
